# COVID-19 Repercussions: Office and Residential Emissions in Pakistan

**DOI:** 10.3389/fpsyg.2021.762746

**Published:** 2022-02-11

**Authors:** Mahmood Rehmani, Madiha Arshad, Munnawar Naz Khokhar, Naveed Anwer, Mohammad Adnan, Rana Tahir Naveed, Huda Irshad

**Affiliations:** ^1^Department of Business Administration, University of Sialkot, Sialkot, Pakistan; ^2^Department of Management Sciences, COMSATS University, Islamabad, Pakistan; ^3^Department of Management Sciences, SZABIST, Larkana, Pakistan; ^4^SBS Swiss Business School, Business and Management Department, Kloten, Switzerland; ^5^Department of Economics and Business Administration, University of Education, Lahore, Pakistan

**Keywords:** environmental challenges, energy management, office emissions, work from home, COVID-19

## Abstract

The purpose of this study is to find empirical evidence on whether work from home or residential emissions reduces office emissions. Based on existing research the study supports that there are short-term effects on office emissions, i.e., carbon emissions do not outshine the long-term effects. The shift from offices to working from home due to COVID-19 regulations meant more people operating from home as maintaining their position in the market was crucial. The potential research area is to understand how this would affect energy usage and carbon emissions. This study has used a before and after mixed approach to collect data from 301 working-from-home employees and 348 top managers who are responsible for monitoring the employees in a work from home setting. Convenience sampling helped collect responses in a timely manner as offices were not allowing visitors and collecting data in person was difficult, so online surveys were conducted. Work from home reduced usage of office equipment, transportation, pollution, etc. The air quality improved considerably but our findings show that the low emissions were only short-lived. This was not a long-term scenario as organizations kept practicing their operations even at home and the emissions stayed in the environment. Future suggestions and implications are also provided. The results give new insights to researchers in the field of sustainability and the environment.

## Introduction

The coronavirus (COVID-19) pandemic has impacted on every field of life globally and has drastically stimulated environmental change. Increased greenhouse gases (GHG) and a high level of carbon emissions have become alarming concerns in recent decades. The upsurge in pollution levels has excessively affected life on earth and have always been a perturbing concern for irreparable and grave damage to the environment and life. Office emissions are among the top-most contributors to environmental pollution. PM_2.5_ refers to fine particles with a diameter of 2.5 such as dust, smoke, and pollen and consists of all liquid or solid particles suspended in the atmosphere. It is the primary reason for smog and, as Zhao et al. (2021) say, industrial and residential emissions were the dominant contributors of particulate matter during COVID-19. GHG emissions are contributed to by CO_2_ and its equivalents such as methane, nitrous oxide, and fluorinated gases. According to WHO (1986), indoor air pollution in workplaces is widely recognized as one of the most serious potential environmental risks to human health. Indoor air contaminants in office buildings like sprays and air fresheners have deteriorated air quality.

Greenhouse gases (GHG) emissions from human activities include the burning of fuels for electricity, heating, and transportation. Transportation requires the burning of gasoline and diesel to release carbon dioxide and greenhouse gas into the environment. Electricity on the other hand is necessary to carry out workplace operations as the higher energy demand is observed due to the work from home setting (Abu-Rayash and Dincer, 2020) and thus adds a major portion to office emissions. The GHG emissions generated by human activity, such as the use of computers, printers, and vehicles, exerts extra pressure on what is otherwise a self-balancing Earth system. CO_2_ is the major contributor to GHG emissions and the aforementioned release the gas into the environment (Srinivasan et al., 2008).

In the aftermath of the pandemic, many businesses that were at risk in the beginning have strategically planned and started to grow by ensuring the observance of regulations advised to reduce the transmission of coronavirus (Waqas, 2021). Many businesses have adopted the work-from-home policy for a major chunk of their workers. There was no other way to keep the businesses from failing. According to Irshad et al. (2021), work-from-home practices significantly and positively affected employee performance during COVID-19. Pakistan is a developing country with a low GDP, so it is better to revise compensation plans and retain high performing employees to prevent unemployment and hiring costs. Organizations are forced to drive their culture of office work in a completely new direction and to do this they have motivated their employees and facilitated them to work from home efficiently (Gallagher, 2020; Waizenegger et al., 2020).

This new trend has had a vivid effect on the environment. On one hand, the reduced commutation to and from offices has reduced fossil fuel consumption and, on the other hand, the usage of office stationery and electricity has also reduced at the workplace. Minimized office crowds, on the other hand, have elevated the thought that this pandemic may cause a reduction in carbon emissions to a decent level by measuring the change in activity as a function of confinement level (Le Quéré et al., 2020). Although the COVID-19 crisis is an international health disaster with serious implications for health and business, it could also enhance the air quality by reducing carbon emissions which will lead to enhanced environmental health (Rupani et al., 2020). This study attempts to understand what the effects of energy usage and carbon emissions would be if the majority of people work from home. On the contrary, this may also shift the burden of office emissions to the residential sector and this shift needs to be recorded as well.

Organization forced confinement and government policies during COVID-19 has led to radical changes in human activities globally. WHO suggested social distancing right away as a preventative measure against the coronavirus. To practice social distancing institutes and offices were closed, exams were cancelled, and the only medium of communication was online (Irshad et al., 2021). According to Sohrabi et al. (2020), the world health organization issued the standard operating procedures to control and minimize the effects of coronavirus. Work-from-home is being widely practiced and it is sweeping away the carbon and GHGs emissions from the environment. Many studies on the other hand emphasized that work-from-home is increasing the electricity requirement of residences. The relative energy intensity of heating and cooling the entire homes of employees rather than a single office suggests that the future of working from home is not as green as one might think based on reduced commuting alone (Cicala, 2020).

Have these government policies and enforced confinements by organizations during this outbreak dramatically altered energy demands? If this were factual, then it would result in tremendous change. It is important to examine and analyze the trend of emissions before and during the pandemic as the results can help to anticipate the major culprits of office emissions and sense the underlying factors involved in it. Comparing the levels before and during the pandemic would help to understand the extent of change in emissions, a possible shift of emissions from commercial to the residential sector, and may result in directing companies to set new trends of working-from-home. Switching to this setting may lead companies to control emissions, cut their costs associated with on-site working, and efficiently benefit from a diverse intellectual, skilled, and knowledgeable workforce (Iftikhar et al., 2021).

The outbreak of this pandemic has affected the world immensely. Many studies and researchers are analyzing its effects on every aspect of human life and nature (social, cultural, socio-economic, wildlife, environmental etc.). As this issue will engrave its imprint on the globe forever, this research study may benefit organizations and policymakers to understand the issues of organizations’ carbon footprint on the environment and environmental issues related to working-from-home.

## The Literature Review

The International Energy Agency reported the impact on energy-related carbon emissions caused by the COVID-19 lockdown. According to this report, global total energy-related CO_2_ emissions in 2020 are expected to fall to 30.6 GtCO_2_, an annual decline of 8%, which is the lowest level since 2010. Such a year-on-year reduction would be the largest ever, six times larger than the previous record reduction of 0.4 GtCO_2_ in 2009 caused by the global financial crisis, and twice as large as the combined total of all previous reductions since the end of World War II (Dafnomilis et al., 2020).

The World Health Organization (WHO) demonstrate a serious concern in a continual rise in pollution. A constant upsurge in GHG and CO_2_ emissions is a foremost concern and is deteriorating environmental health and adversely affecting life on earth. Office emissions contribute a major portion of environmental pollutants. Defining its boundaries is the most challenging and critical aspect due to the diversity of available equipment and variety in operations and environmental conditions. As every organization is different, each requires different setups for their offices. Computers, copiers, printers, fax machines, air conditioners, lighting, and other electronics are essential items of an office. Office equipment is a recognized source of volatile organic compounds (VOCs) and semi-volatile organic compounds (SVOCs) emitted by the paper processed during printing and copying (Mendell et al., 2002; Nielsen, 2005; Destaillats et al., 2008).

### The Carbon Footprint of Change in Commutation to-and-From Offices

Commuters to and from offices use public or private transport every day and contribute to environmental pollution. Traffic congestion increases emissions from vehicles. In many areas, vehicle emissions have become the dominant source of air pollutants, including carbon monoxide (CO), carbon dioxide (CO_2_), volatile organic compounds (VOCs) or hydrocarbons (HCs), nitrogen oxides (NOx), and particulate matter (Zhang and Batterman, 2013).

Forced lockdowns limited the traffic on roads, consequently minimizing the CO_2_ emissions and enhancing the air quality. Many international borders were closed and populations were confined to their homes, which reduced transport and changed consumption. A decrease in daily global CO_2_ emission is recorded by early April 2020 compared with 2019. A recent study analysis shows that for people who commute by car, working from home is likely to reduce their CO_2_ footprint, for those whose travel to the workplace is greater than six kilometers. However, for short car commutes or those done by public transport, working from home could increase CO_2_ emissions due to extra residential energy consumption (Slav, 2020).

### From Offices to Work-From-Home

Coronavirus has radically affected every aspect of human life and economies over the globe. To avoid its spread, many policies and regulations were formed and many countries followed the lockdown strategy. To slow down the rapid growth of coronavirus, people were confined to their homes and the shut down of many activities directed the earth toward a break. Many organizations have shifted workplaces from offices to homes. It is important to note that this enforced work-from-home was entirely different from any pre-COVID-19 remote working scenarios as most of the company’s workforce needed to work from home, not only a few employees or teams (Waizenegger et al., 2020). COVID-19 downgraded many employees as it became difficult to adapt to sudden changes and understand the technology. Fear of losing their jobs kept them motivated and they continuously progressed on their work. This obligated many office workers, working from home, to expense their private life, thus unknowingly increasing their residential emissions. Significant disruptions were made to daily patterns of life as workplaces closed and more time was spent at home. These changes in daily rhythms are reflected in monthly data from utilities around the country, with residential consumption rising by 10% on average, and commercial and industrial consumption falling by 12% and 14%, respectively, during the second quarter of 2020. The rise in residential consumption means that households spent nearly $6 billion on excess electricity from April-July to, 2020 (Cicala, 2020). It is an obligation of every knowledge worker working from home to use a computer system, tabletop printers, stationery items, etc. to accomplish their organization’s demand for work. Irina Slav (2020) also acknowledged in a research study that lockdowns have also affected residential energy demand. Although overall electricity consumption plunged by 20% or more, energy utilities reported increased residential demand because of people spending more time at home. Hourly demand patterns on weekdays resembled those of a normal Sunday.

Reduced commutes to and from offices led to less usage of printers, scanners, and other office equipment and minimized use of paper and electricity, which has substantially lowered CO_2_ emissions from offices. The COVID-19 crisis is an international health disaster with serious impacts on health and business, yet COVID-19 can enhance the air quality by reducing carbon emissions which will lead to enhanced environmental health (Rupani et al., 2020).

Organizations strive to cut down their carbon footprint, thus aiding the environment. Companies control their emissions, i.e., greenhouse gases, which may include water vapor, methane, carbon dioxide, nitrous oxide, ozone, and chlorofluorocarbons (CFCs), for the welfare of the society and adding goodwill to the company. Office emissions may include gases, released from heating and cooling systems, waste (garbage) disposal, ultra-fine particles released from printers and copiers, use of harmful insect killers and air fresheners, and carbon emissions (Rehmani et al., 2020). Defining limits of office emissions is a challenging task as organizations vary in many aspects and a variety of operations and different setups make them unique from one another, such as their HR department.

Remote working is an arrangement of working outside of a traditional setting of an office. This has allowed employees to work while being cost-effective and productive at the same time. Work from home can save money because of less or no commute to and from office, while flexible hours of working allow employees to work comfortably and spend significant time with family, whilst adding value to their income.

Ecological crises emerge when various human behaviors and activities surge and then contribute to the environment. A heuristic explanation determines that changes in human behaviors and activities can lead to a sustainable environment setting to reduce damages. Affordance theory, which interprets environmental behavior from an ecological approach, is shown to be a promising heuristic for systemic behavior analysis (Kaaronen, 2017). Abrupt decisions and forced regulations of government for lockdown during the coronavirus outbreak forced millions of people to work from home. Remote working became the need of the hour to safeguard and protect human lives from a life-threatening virus. This major shift in human activity, following the theory of affordance, became an underlying reason for environmental changes. Thus, the affordance theory portrays that reduced usage of office equipment, e.g., printers, copiers, computers, and heating and cooling systems, plus fewer commuters to and from offices either reduced or shifted emissions from office to residential. Transferring employees from the office may also shift its emission level to the residence. So, the research question is can work from home reduce office emissions or shift emissions from the commercial to the residential sector? This study is designed to find the answer to this question so that further advanced research could reveal the critical managerial implications of work-from-home.

## Methodology

To understand the shifts of emissions from offices to residences, we used short surveys to understand the impact of office and residential emissions. A short questionnaire was formed for knowledge workers who worked in an office environment before Covid-19 and then were forced to work from home, and the second questionnaire was formed for industry CEOs and policymakers to comprehend the increase or decrease in office emissions. Both the questionnaires included a mix of open and close-ended questions. Somequestions were measured by a Likert scale.

For office emissions, respondents were asked to share details about their city and gender, the industry they worked in, employment status, safety measures taken, number of employees working from home, months since working from home, aids provided (computer system, printer, internet connection, and stationery), problems they were facing, and what form they submitting work in were (soft or hard). Using a 5-point Likert scale, respondents filled the survey based on work from home and office regarding photocopiers, air conditioners, heating systems, stationery items, air freshener, insect killer, electricity, and office wastage. Moreover, they were also asked about the changes in their bills and what work setting they preferred (work from home or office).

For home or residential emissions, a few questions were similar such as industry, city, gender, level of employment, safety measure, number of months since work from home, aid provided, problems faced during work from home, and what form they were submitting work in (soft or hard). Using a 5-point Likert scale, respondents chose based on their convenience for completing deadlines working from home. A few questions from the questionnaire are as follows: Did you use your personal computer to complete your assigned tasks, To what extent did you compensate your personal work for your office work, Did you use your personal printer for office work, Did you purchase your own stationery items (paper, pens etc.) for office work, Did you compensate your family time to complete office work, Usage of home air conditioner (AC) when you work in office and home, Usage of home heating systems when you work in office and home, What were your residential electricity bills when you work from office and home, What were your residential gas bills when you work from office and home, What was your petrol consumption when working from office and home, and What was your home waste (garbage) when working from office and home. And lastly, the respondents were asked if they preferred working from home or office. The data was then analyzed with the collaboration of secondary sources to comprehend how pandemics can environmentally affect change in the long run. According to the short-term effects of COVID-19 in reducing carbon emissions were considerably high but were short-lived.

Due to COVID-19, almost all departments of the country were closed, and most of them worked from home. In this context, we recruited the population by virtually distributing both questionnaires over the country using convenience sampling and a mixed approach. The population includes all types of offices in Pakistan using heating and cooling systems, printers, copiers, and computer systems. Responses of 301 knowledge workers who were forced to work from home and 348 CEOs and policymakers who enforced employees to work from home were received through an online survey.

Thematic analysis was conducted to analyze the responses due to the qualitative nature of the data (Nowell et al., 2017). First, the familiarization with data is done. Each response is examined to gain familiarity with the trend of emissions. Then themes were identified and reviewed iteratively. Finally, the theory of affordance by Gibson (1977) was used to inspect findings through its perspective. For analyzing the increase or decrease of emissions and to understand their shifts, it is important to understand trends of usage of emission-causing objects during and before COVID-19.

## Results

Survey results showed that computer systems showed a 20% decrease in office usage when employees were working from home and a 15.65% increase in computer systems used during the employee’s work from home. This was because the company was bound to provide a computer system to their employees to aid them during work from home. This shows a shift of VOC, SVO’s and other matters from the office sector to home.

Printers, on the other hand, are important equipment in an office. During and before COVID-19, 33.62% of printer usage remained the same. This is because employees submitted their work in soft copies so the emissions did not increase or shift from commercial to the residential sector.

Copier machines were mostly used in the office and minimum to zero usage is shown in the residential sector, evidently showing that the emissions from the copier machine at the office remained constant as it was before COVID-19. Therefore, there is no shift toward the residential sector.

Air conditioners used in the office decreased from 32.75 to 27.83%. It shows the reduction in emissions from air conditioners. Employees reported no increase in usage while they work from home, as their family was already using the equipment before the pandemic, and the same use was in practice during the pandemic.

Heating systems do not show a notable change in emissions. The usage remains the same in the office, as most of the offices had not installed heating equipment for employees’ offices. Employees’ report the same level of usage at home, as their family was already using the heating system before and during the outbreak of COVID-19.

Stationery items show an increase from 36.81 to 37.68%. The office budget of stationery items increased because companies were bound to aid every individual employee with stationery items to help them work from home, so it did not increase the wastage at the office, but shifted it to the residential sector as the stationery consumed was at the residential sector.

Air freshener shows a 13.04% decline in use when employees work from home. Employees use of air fresher at their home is at a minimum level. Thus, it has reduced the usage at the office level, but it has not shifted to the residential sector.

**FIGURE 1:**
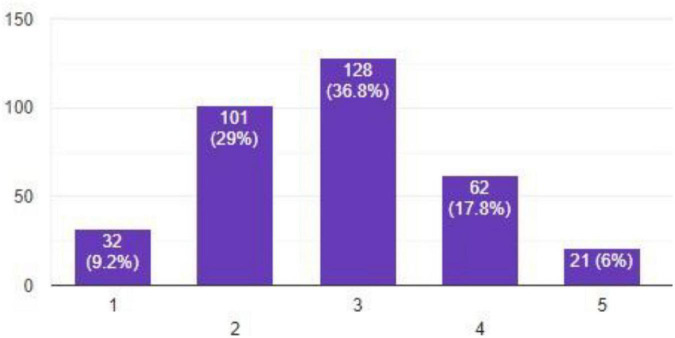
Change in electricity and gas bills.

Petrol consumption of employees who commuted to and from offices reduced from 42.09 to 35.02%, thus adding less pollution to the environment. Electricity and gas bills play a major role as shown in Figure 1 and are important in analyzing overall emissions in commercial and residential sectors. Change in electricity and gas bills is shown in the following bar chart.

**FIGURE 2:**
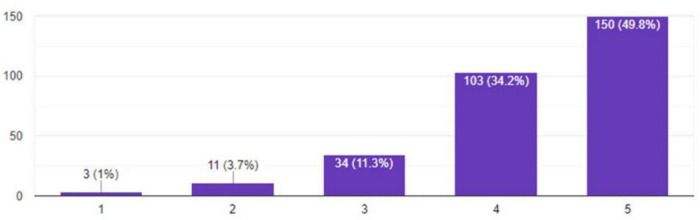
Bills during remote working.

**FIGURE 3:**
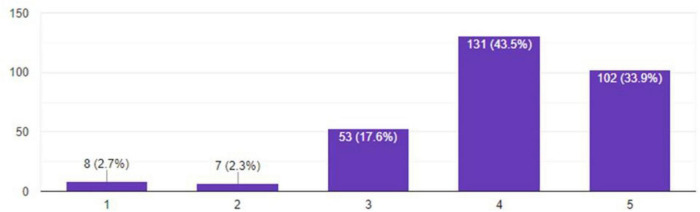
Bills during on-site working.

A maximum number of respondents report that electricity and gas bills stayed at a moderate level of consumption. Employees on the other hand report bills before remote working were 33.09%, which during the pandemic increased to 49.08%, showing a 15.99% increase. Figures 2, 3 show the bills during remote and on-site working.

Working from home is government-forced confinement and organization’s policies were imposed during the COVID-19pandemic. Comparing responses of home and office emissions before and during the coronavirus pandemic allowed for comparing the use of computer systems, printers, copiers, stationery items, insect-killing and air fresher sprays, utility bills, and wastage (garbage). However, it is important to consider how the situation was different from the pre-COVID-19 situation. First, the knowledge workers were forced to work from home as per regulations of the government. The decision was not by choice, but it was an obligation. Second, a large number of employees of organizations were working from home. Third, because of the risk of the virus employees were concerned about themselves and their family’s health. This increased the stress of employees. Keeping all the above factors in focus, it is comprehensible that employees working from home in the pre-COVID-19 situation were not as common than during the pandemic as the whole workforce was now working remotely.

The demand for computer systems, strong net connections, printers, and stationery items surged for employees working from home. Many organizations aided their employees by providing them with the required equipment. Still, many of these were purchased or personal items were bought in use for the working of the organization. Looking at the graph generated by responses of the employee’s shows that net connectivity and stationery items are the highest bars representing aid provided by organizations to their employees. This thus shifted emissions from office to home.

Employees reported that there was a moderate increase in utility bills of electricity and gas, as well as a slight surge in garbage and waste. This portrays that it did not add to home emissions. Emissions of office were reduced to some extent as computer systems, copiers, printers, and heating and cooling systems were somehow in use as most of the work submitted was in soft copy and heating and cooling systems were in use as top management were on-site monitoring employees by following SOPs. During the peak of the global confinements in the first quarter of the year, daily emissions were about 17 per cent below last year, according to research published this week in Nature Climate Change.

Looking through the lenses of affordance, the functional affordance of working of an organization remained the same before and during the lockdown, while the decrease in office emissions was subject to the short-term outcome, as the organizations need to grow and generate profits, and they demand the continuation of their operation. Whereas the loss of environmental affordance of the formal setup of working from the office and using the pieces of equipment of that setup obligate to portray the shift of emissions from office to residential. However, this shift was limited. According to Borunda (2020), a reduction in office emissions during the pandemic is not something to celebrate. Instead, the only way to do that is to bring about technological, behavioral, and structural change in organizations. The objective of this study included understanding how energy usage and carbon emissions affect the environment if a major chunk of the working-class continued working from home. And whether these emissions from before and after the pandemic can provide evidence for underlying factors that contribute to office emissions. The graphs for bills according to the respondents show that there was no drastic change in the bills. There was a rise in bills working from home as compared to working at offices.

## Conclusion

Results show a decrease in office usage but an increase in computer systems during work from home. Printers were not used much as data was exchanged through emails. There were no changes in the usage of copier machines. Air conditioners and heating systems were already in the houses of employees so there was no increase in their usage. Stationary items increased, shifting the wastage to the residential sector and air freshener usage decreased fairly. Most importantly, the vehicle usage lowered, resulting in no addition to pollution. Working from home increased the electricity and gas bills as the whole workforce worked remotely. As mentioned above, good laptops, internet connection, printers, and stationary items were in more demand than before which caused the office emissions to shift to residential. This reduction in office emissions was subject to a short-term period and no enormous changes in percentage decreases were noted. Employees working from home did not find much increase in home emissions as they utilized the pre-COVID-19 equipment already present in their homes. The study shows how energy usage and carbon emissions affect the environment in a work-from-home setup. Confining employees to work from home does not add to environmental emissions and a shift may occur in the short-term but it does not apply to long-term scenarios.

## Future Recommendations

This study opens new horizons for environmental researchers focusing on reducing carbon emissions. Furthermore, this study provides new dimensions to the corporate sector that show sensitivity toward the deteriorating environment and adopt sustainable strategies to reduce the impact of their business activities on the surrounding environment. National-level data from utility companies and patterns of energy usage in commercial and residential areas during the lockdowns observed in the wake of COVID-19 could further explain the overall impacts and could be an interesting topic of study for future researchers. This study also calls for in-depth research on the impacts and efficacies of shifting workplaces from offices to homes permanently. Practical implications include using this study as a foundation for finding evidence on the short-term impacts of office emissions in Pakistan by using a quantitative approach. The findings of this study have theoretical implications as the affordance theory states that the world is not only perceived in terms of what we see but for what the environment offers us; the possibility of something different, such as potential variables as technological incompetence, lack of structure, inexperience, etc., can be a reason for office emissions. Future research should find such causal relationships between variables and emissions. This study has a few limitations; open-ended questions increased the biasness and vagueness in results. Future studies should use close-ended structured questions for better results and the study can be conducted to analyze the shift of emissions from large office setups that may have been subject to share on-site equipment’ emissions, whereas, employees performed their tasks individually at their homes and this separate working may have added more to environmental emissions.

## Data Availability Statement

The original contributions presented in the study are included in the article/supplementary material, further inquiries can be directed to the corresponding author.

## Ethics Statement

The study did not involve human subjects. Data was collected through survey questionnaires and before floating the questionnaire consent was obtained from each participant. The questionnaire included a statement that we will retain anonymity and confidentiality of the collected data and use the data for any academic purposes only. The participants filled the questionnaires voluntarily and participated in this study with consent.

## Author Contributions

MR, MAr, and MNK contributed to conception and design of the study. They also reviewed literature. NA and RTN added rigor and purposiveness to the article. MAr wrote the first draft of the manuscript. HI contributed as the coordinator and corresponding author. MAd helped proofread the article. All authors contributed to the manuscript revision, read and approved the submitted version.

## Conflict of Interest

The authors declare that the research was conducted in the absence of any commercial or financial relationships that could be construed as a potential conflict of interest.

## Publisher’s Note

All claims expressed in this article are solely those of the authors and do not necessarily represent those of their affiliated organizations, or those of the publisher, the editors and the reviewers. Any product that may be evaluated in this article, or claim that may be made by its manufacturer, is not guaranteed or endorsed by the publisher.
